# Humidity trends imply increased sensitivity to clouds in a warming Arctic

**DOI:** 10.1038/ncomms10117

**Published:** 2015-12-10

**Authors:** Christopher J. Cox, Von P. Walden, Penny M. Rowe, Matthew D. Shupe

**Affiliations:** 1 Cooperative Institute for Research in Environmental Sciences, University of Colorado, Boulder, Colorado 80309, USA; 2 NOAA Earth System Research Laboratory, Boulder, Colorado 80305, USA; 3 Department of Civil and Environmental Engineering, Washington State University, Pullman 99164, Washington, USA; 4 Department of Geography, University of Idaho, Moscow 83844, Idaho, USA; 5 Departamento de Física, Universidad de Santiago de Chile, Santiago 9170124, Chile

## Abstract

Infrared radiative processes are implicated in Arctic warming and sea-ice decline. The infrared cloud radiative effect (CRE) at the surface is modulated by cloud properties; however, CRE also depends on humidity because clouds emit at wavelengths that are semi-transparent to greenhouse gases, most notably water vapour. Here we show how temperature and humidity control CRE through competing influences between the mid- and far-infrared. At constant relative humidity, CRE does not decrease with increasing temperature/absolute humidity as expected, but rather is found to be approximately constant for temperatures characteristic of the Arctic. This stability is disrupted if relative humidity varies. Our findings explain observed seasonal and regional variability in Arctic CRE of order 10 W m^−2^. With the physical properties of Arctic clouds held constant, we calculate recent increases in CRE of 1–5 W m^−2^ in autumn and winter, which are projected to reach 5–15 W m^−2^ by 2050, implying increased sensitivity of the surface to clouds.

## Abstract (web summary)

The cloud radiative effect (CRE) in the Arctic plays an important role in the amount of infrared radiation that reaches the surface. Here, the authors show that interplay between temperature and humidity controls CRE through competing influences in two semi-transparent wavelength ranges.

## Graphical abstract

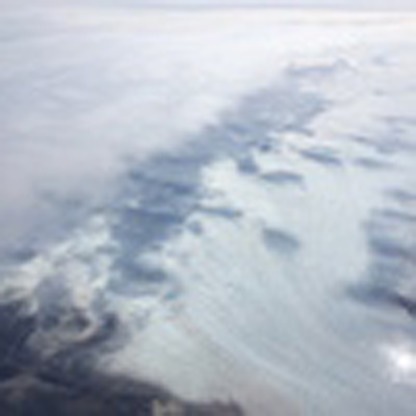

Amplified warming of the Arctic and coinciding decreases in sea ice are driven in part by perturbations to the surface radiation budget^1^,^2^,^3^,^4^,^5^. The infrared cloud radiative effect (henceforth, ‘CRE') at the surface can be 50–100 W m^−2^ (refs 6, 7, 8, 9, 10); however, the system is sensitive to comparatively small changes. In fact, observed decadal trends in sea ice could be forced by a perturbation of just 1 W m^−2^ (ref. 11). Variability in surface cover (due to variability in snow/ice cover) modifies albedo, which is a control on shortwave CRE that is not directly due to cloud properties^12^, that is, clouds cool the surface relative to clear skies by reflecting sunlight back to space; however, this cooling is minimized over ice-covered surfaces because the albedos of the surface and cloud are similar^13^. Nevertheless, in spite of changes in surface cover in the Arctic, it is likely that cloud feedbacks at high latitudes are dominated by infrared radiation^12^.

Globally, infrared emission from clouds reaches the surface primarily through the ‘atmospheric window' (AW; defined here as 7–14 μm) where absorption by atmospheric gases is relatively low^14^. However, in dry regions, such as the Arctic, the far-infrared (FIR; 16–40 μm) is also semi-transparent, and variability in clouds^14^, water vapour^15^,^16^ and surface emission^16^,^17^ all contribute substantially to energy exchanges between the surface and space. Since the infrared radiance received at the surface depends nonlinearly on absorption and emission from clouds and water vapour, both of which depend on temperature, atmospheric feedbacks and processes implicated in Arctic warming^1^,^2^,^3^,^4^,^5^,^12^ are not easily disentangled. Increases in water vapour are reported in the Arctic^18^,^19^ and projected by climate models^20^, but temperature–humidity relationships in the Arctic are distinct from lower latitudes^21^, making it difficult to project the effects of atmospheric change on the surface in the presence of clouds. Similar to the way that changes in surface cover influence the shortwave CRE, temperature and humidity exert an influence that is independent of cloud properties. Therefore, variability in CRE due to variability in the atmospheric state must be addressed.

Here we present an analysis of spectrally resolved CRE using surface-based measurements from three Arctic observatories. Interplay between temperature and humidity is shown to control CRE through competing influences in two semi-transparent wavelength ranges (7–14 and 16–40 μm), stabilizing CRE at constant relative humidity for temperatures characteristic of the Arctic (∼230–280 K). Using reanalysis and climate model data sets, we find that, because of this mechanism, increases in temperature and precipitable water vapour (PWV) in the Arctic are likely resulting in increases in CRE that are independent of changes in cloud properties.

## Results

### Observations of the CRE relation with temperature and PWV

**Figure 1:**
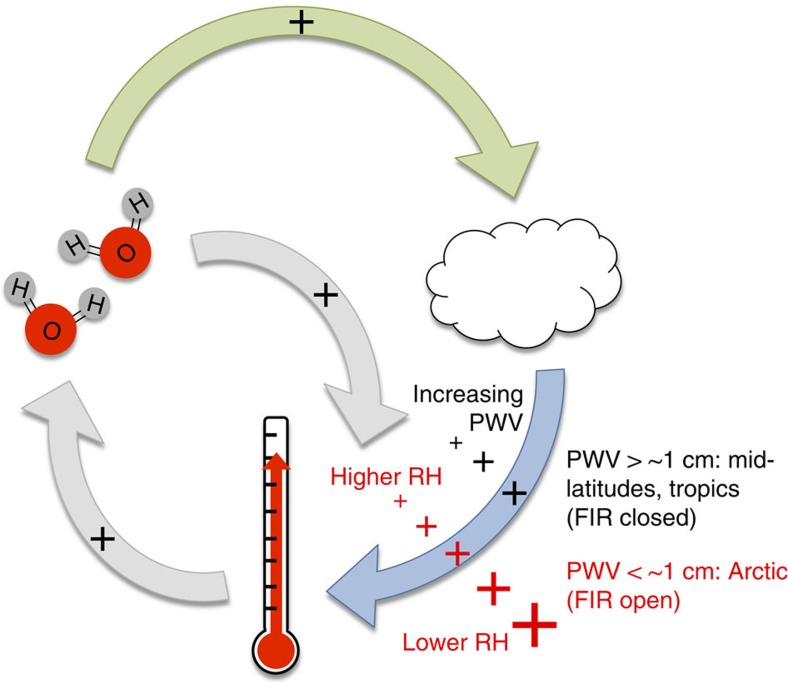
Conceptual diagram of Arctic feedbacks relevant to CRE. Conceptual diagram of Arctic feedbacks involving surface temperature, atmospheric water vapour and clouds. The grey arrows represent the ‘water vapour feedback'^21^. The green arrow represents the subsequent change in cloud properties; for example, an increase in the amount of clouds^23^ increases the surface temperature via increased CRE (blue arrow). The magnitude of the black and red plus (+) symbols represents perturbations to the CRE feedback; note that the total change in CRE is influenced by changes in cloud properties as well. Increasing atmospheric water vapour *dampens* the increase in CRE feedback from changing cloud properties, indicated by decreasing black ‘+' symbols. When the FIR is open, as relative humidity (RH) decreases, the CRE feedback increases (red ‘+' symbols increasing), as described in this study.

**Figure 2:**
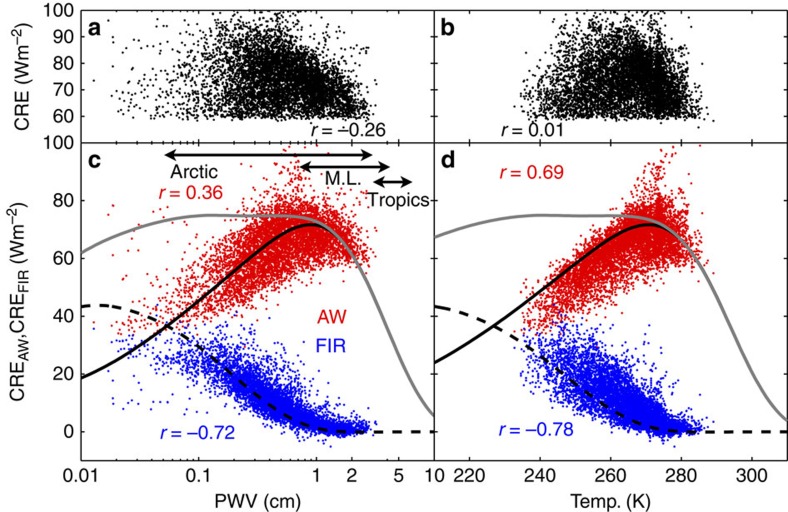
CRE in the FIR and the atmospheric window. (**a**) Observations of 3-hour averages of downwelling infrared CRE from Barrow, Alaska; Eureka, Canada and Summit Station, Greenland, plotted as a function of PWV. Only CRE>60 W m^−2^ are shown to highlight clouds that are optically thick. (**b**) Same as **a**, but plotted as a function of near-surface air temperature. (**c,d**) are similar to **a**,**b**, but are separated into spectral components from the atmospheric window (CRE_AW_, red points) and the far-infrared (CRE_FIR_, blue points). For reference, the arrows in **c** are the approximate ranges of PWV for the Arctic, mid-latitudes (M.L.) and Tropics. Curves in **c**,**d** are radiative transfer calculations of CRE_FIR_ (dashed black), CRE_AW_ (solid black) and their sum CRE=CRE_FIR_+CRE_AW_ (grey).

In a well-known positive feedback, atmospheric water vapour increases with temperature, and because water vapour is a strong greenhouse gas, this leads to more water vapour emission to the surface and thus further warming^22^ (grey arrows in Fig. 1). Increases in water vapour also lead to increases in cloud cover and/or optical depth, both of which feedback to increased warming through additional CRE (for example, ref. 23; green and blue arrows in Fig. 1). However, absorption and emission from water vapour between the cloud and the surface mask the radiance from the cloud. In particular, cloud emission must be transmitted through the atmosphere below the cloud to reach the surface and have an impact. Thus, CRE should decrease as humidity increases concurrently with temperature. Indeed, smaller CREs are noted for the wet season in the tropics as compared with the dry season, due in part to differences in humidity^24^. A similar relationship likely occurs at mid-latitudes, although the effect has not been isolated from the seasonal cycle in cloud fraction^25^. This relationship plays out as decreases in the cloud feedback though each iteration of the loop in Fig. 1 (black ‘plus' symbols in the lower right of the diagram signify increases in humidity with time). However, for the Arctic, we find that CRE is poorly correlated with observed surface temperature and PWV (Fig. 2a,b), suggesting that the behaviour of the cloud feedback is different in the Arctic compared with lower latitudes. Our analysis begins with an examination of the observed CRE in the Arctic with the objective of isolating the influence of the atmospheric state from the influence of variability in cloud physical properties.

CRE is defined as the difference between downwelling infrared flux from clouds and atmospheric gases together and that from gases alone,


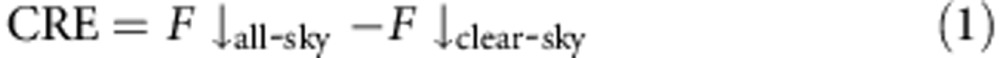


In this study, CRE is calculated from the perspective of the surface. At the surface, CRE differs from cloud radiative forcing (CRF) in that CRE is for the downwelling infrared component only, rather than the net flux, but they are similar because the upward component of CRF is small. To isolate how temperature and humidity influence CRE, it is instructive to hold cloud physical properties constant, either by subsetting observations that represent samples of optically thick clouds, or by performing radiative transfer calculations for clouds with fixed optical depth. The main assumption that is made in either case is that tropospheric temperature covaries with cloud temperature.

Three-hour averages of CRE at stations representative of different regions of the Arctic—Barrow, Alaska; Eureka, Canada and Summit, Greenland—are derived using observations^6^,^7^. The observed values of PWV at these locations span a large range from less than 0.1 cm in winter at Summit^26^ to ∼2 cm in summer at Barrow^6^. Like much of the far-western Arctic, Barrow is relatively moist with a high incidence of optically thick clouds^6^,^9^,^10^. Eureka is representative of the northern Canadian archipelago, a drier region with fewer optically thick clouds than Barrow^6^,^27^. Summit is a high-altitude station on the Greenland ice sheet that is extremely dry^26^. To assess how variability in CRE is modulated by temperature and humidity contributions in the FIR and AW, CRE is calculated here as partial-band fluxes^14^,^28^ such that CRE≈CRE_AW_+CRE_FIR_.

**Figure 3:**
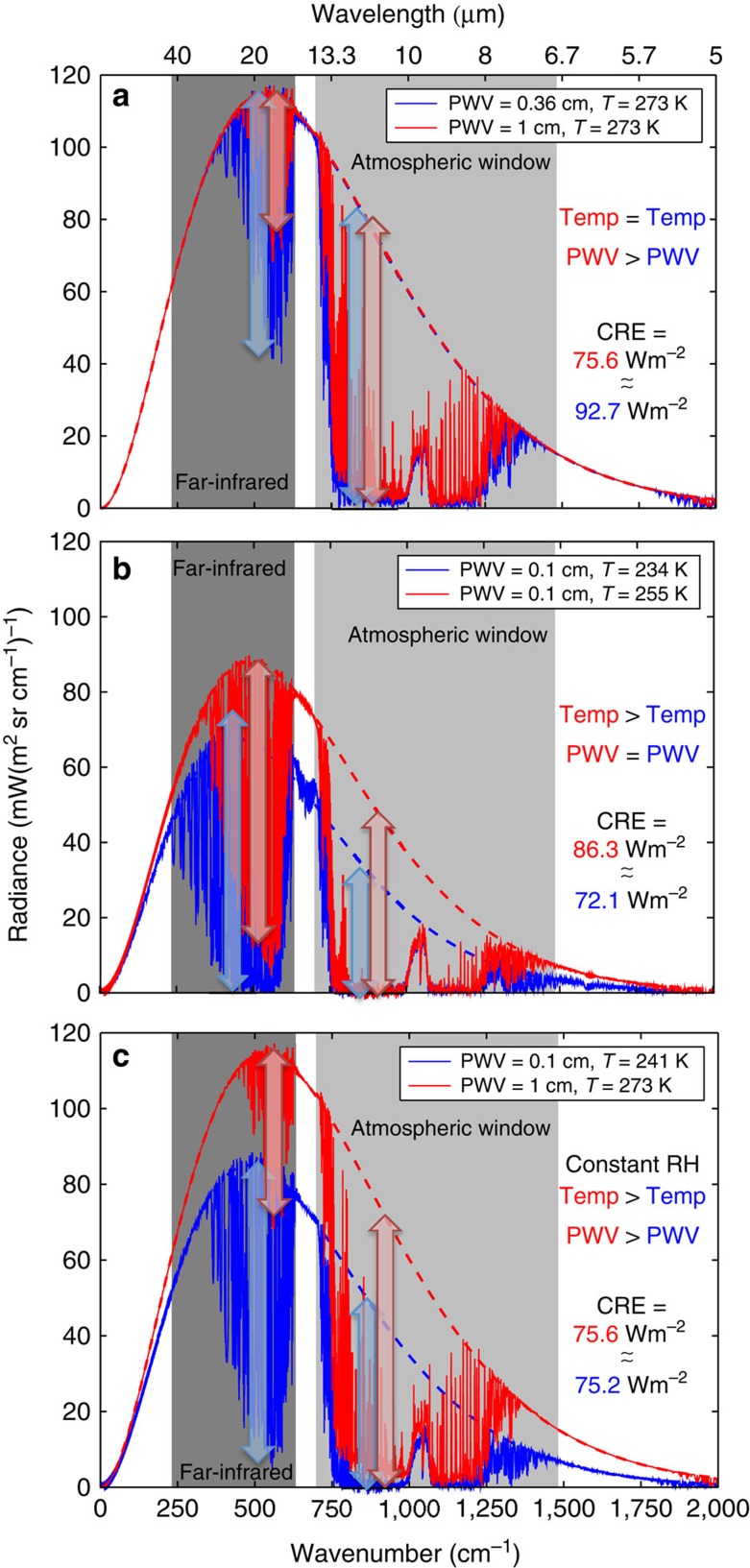
Examples of infrared spectra. Examples of ‘clear-sky' infrared spectra calculated from profiles of temperature and humidity measured by radiosoundings at Barrow, Alaska (red) and Summit Station, Greenland (blue). The dashed lines are Planck functions corresponding to the near-surface air temperatures for the respective cases and represent hypothetical, optically thick clouds. The FIR and AW spectral regions are shown as dark-grey and light-grey shaded regions. The downwelling infrared CRE is the spectral integral of the difference between the curves (dashed minus solid for any case). (Note that because these spectra are for zenith views, the flux (in W m^−2^) is obtained by integrating the radiance over the hemisphere and over wavenumber (frequency) from 0 to 3,000 cm−1 (ref. 6)). The vertical arrows indicate conceptually the magnitude of the CRE in each window. (**a**) Spectra from 12 July 2012 (Summit) and 8 May 2008 (Barrow): similar near-surface air temperatures, but different PWV. The CRE is larger at Summit because of additional CRE in the FIR associated with the low PWV. (**b**) Spectra from 7 May 2011 (Summit) and 12 March 2007 (Barrow): similar PWV, but different near-surface air temperatures. The CRE is larger at Barrow because of additional CRE in the AW associated with the higher temperature. (**c**) Spectra from 21 April 2011 (Summit) and 18 May 2008 (Barrow), where the conditions approximate the Clausius–Clapeyron parameterization in Figs 4 and 6. These cases represent both the cold/dry and warm/moist limits of the range of conditions. The total CRE (CRE_FIR_+CRE_AW_) in **c**, represented conceptually by summing the arrows, is similar between the two cases due to the compensating effects described in the main text.

To understand the apparent lack of correlation in Fig. 2a,b, we examine observed Arctic CRE_FIR_ and CRE_AW_, which are plotted against PWV in Fig. 2c and temperature in Fig. 2d (red and blue dots). Over the range of Arctic conditions, CRE_AW_ increases with temperature and PWV while CRE_FIR_ decreases. To put these measurements in context, we simulate CRE using a radiative transfer model for a wider range of surface temperatures and PWVs than those typical of the Arctic (black and grey curves in Fig. 2). CRE is simulated by parameterizing PWV as a function of surface temperature; this is performed by fixing the shape of the temperature profile and scaling it to fit the surface temperature and by using a fixed relative humidity profile. When CRE is plotted for the wider range of temperatures and PWVs, we see that CRE_FIR_ and CRE_AW_ exhibit similar behaviour (that is, both increase, peak and then decrease), but are shifted in temperature and PWV. This shift occurs because of differing water-vapour absorption coefficients, which are larger in the FIR relative to the AW. This is not a surprising result, but what is interesting is the compensation of the two spectral regions when summed (grey line Fig. 2c,d). This compensation obscures the dependence on temperature and humidity between ∼230 and 280 K, and, thus, explains the lack of correlation in CRE shown in Fig. 2a,b. These compensating flux variations are unique to the temperature and humidity ranges observed in the Arctic. Conversely, CRE increases with temperature below ∼230 K and decreases above ∼280 K. Examples of individual infrared spectra that illustrate this spectral compensation are depicted in Fig. 3.

**Figure 4:**
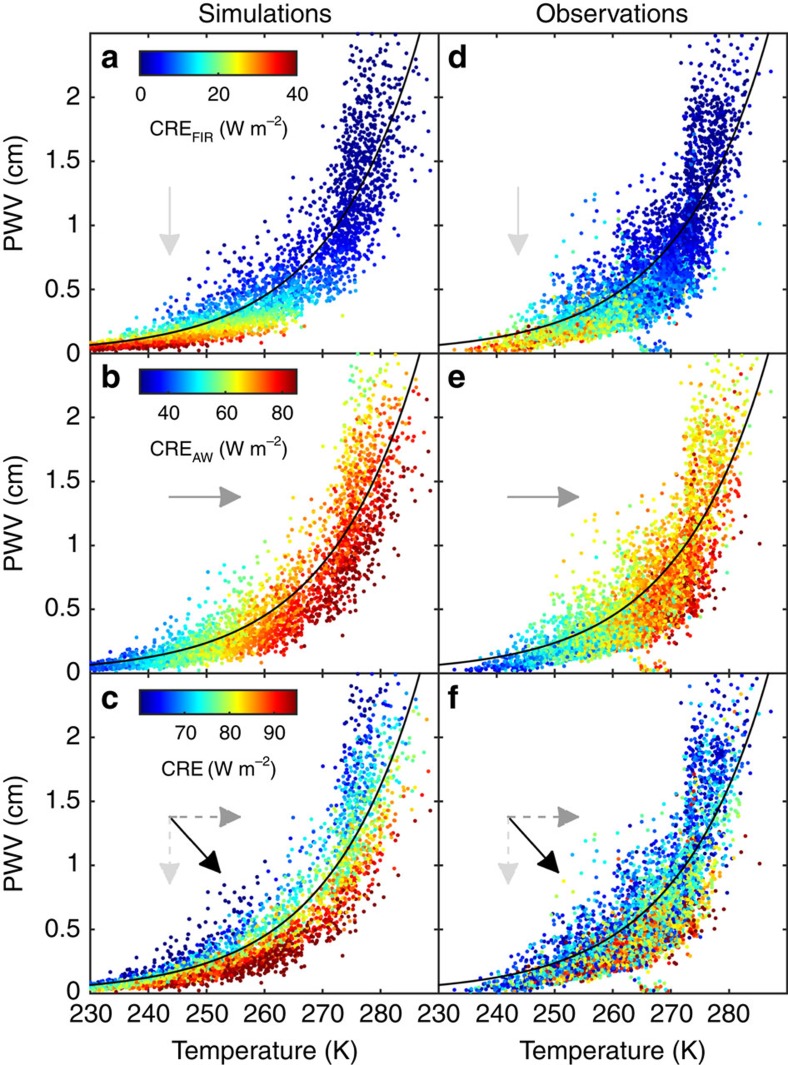
Simulated and observed CRE as functions of temperature and PWV. Simulations (left panels (**a–c**)) and observations (right panels (**d–f**)) of the downwelling infrared CRE in the far-infrared (top panels (**a,d**)), atmospheric window (middle panels (**b**,**e**)) and the total CRE (bottom panels, (**c,f**)). The simulations are from radiative transfer calculations using observed profiles of temperature and humidity acquired by radiosoundings at Barrow, Alaska and Eureka, Canada. The black line is the Clausius–Clapeyron relationship for near-surface air temperature and PWV following ref. 29 and assuming a relative humidity (with respect to liquid) of 100% and a scale height of 3 km. The observed values are data from Barrow and Eureka, but only for clouds with large optical depths (CRE>60 W m^−2^). As a visual aide, arrows show the direction in the temperature–PWV space of increasing CRE, also shown as coloured symbols. Note that the colour scale changes for each row.

To investigate the consequences of this compensation using an idealized framework, radiative transfer calculations are performed using radiosoundings acquired at Barrow and Summit. CRE_FIR_ and CRE_AW_ are calculated based on observed temperature and humidity profiles with model clouds inserted randomly at 0, 1, 2 or 5 km. The modelled values of CRE (CRE_FIR_, CRE_AW_ and CRE) for optically thick clouds (optical depth, *τ*=10) are shown in the left panels of Fig. 4; observed values (explained below) are shown in the right panels for comparison. A parameterization of the Clausius–Clapeyron relationship^29^ is also plotted for reference (black line). Note that CRE is expressed here on a colour scale as a function of both surface temperature and PWV. The modelled CRE_FIR_ (Fig. 4a) varies between 0 and 40 W m^−2^ with a strong dependence on humidity and low sensitivity to temperature. Conversely, CRE_AW_ (Fig. 4b), which ranges from 40 to 85 W m^−2^, is sensitive primarily to temperature. Owing to the compensation described previously, when summed, CRE (Fig. 4c) has values of constant flux that closely follow the Clausius–Clapeyron relationship. Thus, temporal or spatial variations in temperature and/or PWV within the Arctic temperature range do not change CRE as long as the variability is consistent with the Clausius–Clapeyron relationship. However, deviations from this relationship will either increase or decrease CRE over a range of ∼40 W m^−2^ at a given temperature. This behaviour is consistent in modelled CRE for clouds with smaller optical depths, although the magnitudes of the fluxes are smaller (Supplementary Fig. 1).

Observed CREs at Barrow and Eureka, shown on the right column of Fig. 4, agree well with the modelled values in the left column and use the same subset of approximately optically thick clouds shown in Fig. 2. Both the CRE values (colours) and the variations of CRE with temperature and PWV (colour distributions) are quite similar between the model and observations, suggesting that the idealized framework is a reasonable representation of the Arctic.

The results in Fig. 4 explain some of the observed variability in Arctic CRE observed in other studies (for example, ref. 9). For example, the annual cycle in temperature and humidity at Barrow results in variability in the maximum potential CRE of ∼70–90 W m^−2^ with peaks in spring and autumn. As mentioned above, clouds are opaque in the simulations, whereas the true annual cycle in CRE depends also on variations in cloud properties (cloud fraction, optical depth, microphysics and so on). The results of ref. 9 include cloud-property variability and show that the actual peak in infrared CRF occurs in August at Barrow, coinciding with the annual cycle in cloud fraction. Our results are complementary to ref. 9 in that they suggest greater *sensitivity* in the infrared to clouds in the transition seasons (spring and autumn) compared with summer or winter at Barrow, but they do not include the effect of seasonal changes in cloud fraction.

**Figure 5:**
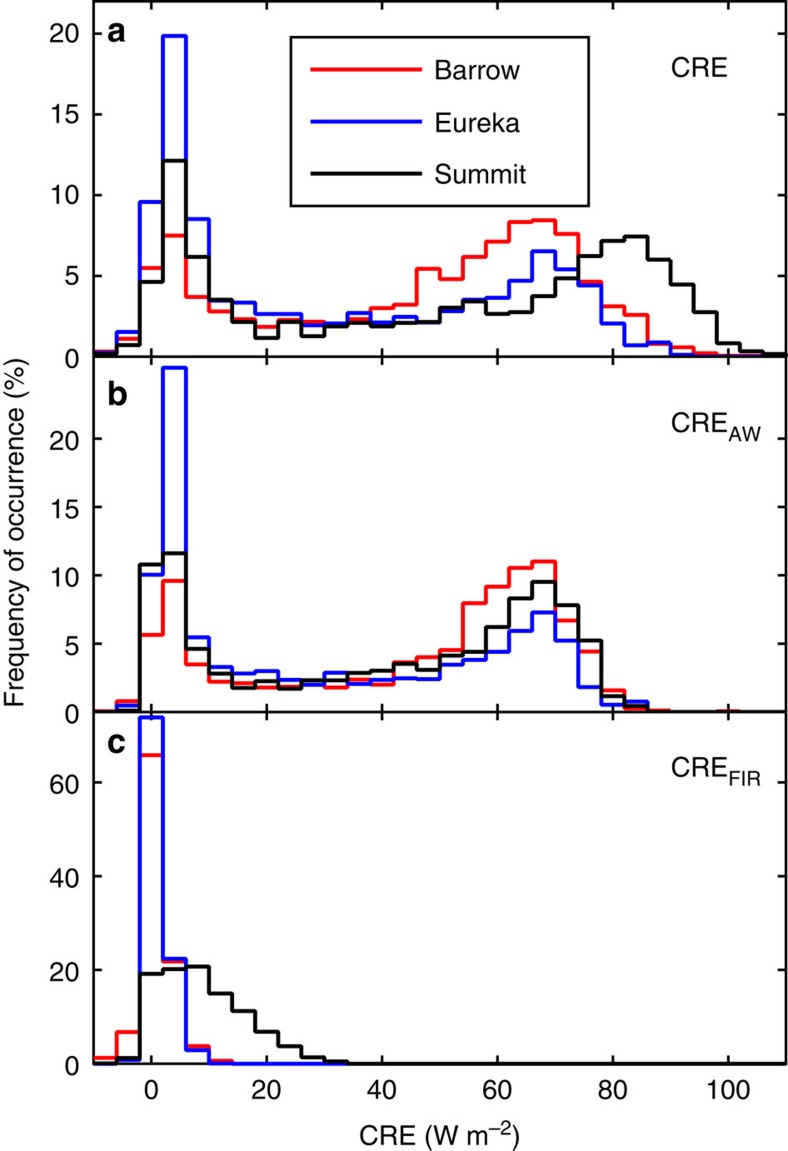
Observed distributions of CRE in summer. Probability distribution functions of 3-hour averages of (**a**) CRE, (**b**) CRE_AW_ and (**c**) CRE_FIR_ in summer (June–July–August) from observations at Barrow, Alaska; Eureka, Canada and Summit Station, Greenland. The bin size is 4 W m^−2^.

Distributions of CRE for all-sky conditions in summer (JJA) at all three stations are bimodal, with one mode representing clear skies (CRE ∼0 W m^−2^) and another representing optically thick clouds (CRE>60 W m^−2^, representative of the subset of clouds shown in Figs 2 and 4; Fig. 5a), similar to distributions reported by others^6^,^7^,^30^. The mode representing optically thick clouds is ∼10 W m^−2^ higher at Summit compared with the other stations. These higher CRE values were, for example, an important driver of widespread surface melting of the ice sheet in July 2012 (ref. 8). Elevated CRE at Summit is due to enhanced CRE_FIR_ (Fig. 5c), rather than CRE_AW_, which is similar at all stations (Fig. 5b).

**Figure 6:**
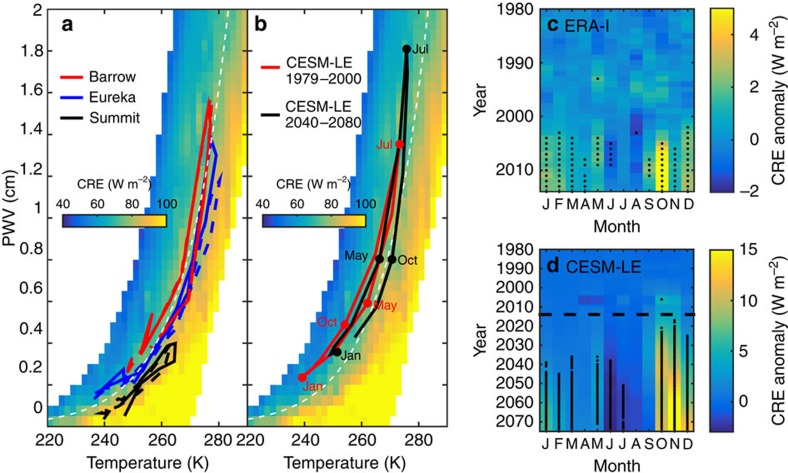
Recent and projected changes in CRE associated with changes in humidity. (**a**) Downwelling infrared CRE as a function of observed near-surface air temperature and PWV for optically thick clouds at Barrow (red), Eureka (blue) and Summit (black). These observations are from the same time period as the CRE calculations in Fig. 5; the dashed lines are for averages over all times, while the solid lines are for averages over times when CRE>60 W m^−2^ (optically thick clouds). (**b**) Similar to **a**, but the red line are CESM-LE monthly mean values from 1979 through 2000, while the black line is for 2040–2080. The CRE surfaces in **a,b** are the values from Fig. 4c interpolated to a T-PWV grid (2 K by 0.05 cm). The white dashed lines in **a,b** are the Clausius–Clapeyron relationship for near-surface air temperature and PWV from ref. 29, assuming a relative humidity (with respect to liquid) of 100% and a scale height of 3 km. (**c**) Estimated anomalies in CRE determined by mapping monthly mean ERA-Interim 2-m air temperature and PWV on the CRE surface in **b**. (**d**) is similar to **c**, but for the CESM-LE historical (1979–2005) and projected (2006–2080) data sets. Data in **c,d** are 5-year running means averaged over the far-western and Eurasian Arctic Ocean domain (north of 70N and 70–220E). Anomalies are calculated using 1979–2000 as a baseline and black dots represent differences between the mean of the 5-year sample (centred on point) and the baseline that are statistically significant (*P*<0.05).

Observed monthly mean temperature and PWV from the stations are plotted in Fig. 6a over the idealized CRE from Fig. 4c, which is interpolated to a surface. The figure shows that differences in the peak of the upper mode of CRE in Fig. 5a are because of deviations from contours of constant CRE, that is, deviations from the Clausius–Clapeyron relationship (dashed white curve in Fig. 6a). Indeed, the conditions at Barrow and Eureka lie close to the Clausius–Clapeyron line, while the conditions at Summit are at lower PWVs relative to temperature (falling below the dashed white line in Fig. 6a). Modelling CRE as in Fig. 2, but incrementally increasing the height of the surface, indicates that station altitude can explain the lower PWV relative to temperature, and thus the higher CRE observed at Summit.

### Projected changes in Arctic CRE

Output from a reanalysis product and a climate model is now used to provide a conceptual understanding of how future changes in the Arctic system might have an impact on its sensitivity to CRE. Using the same methodology to create Fig. 6a, trends in CRE are estimated using the monthly mean temperature and PWV from a reanalysis product and a climate model. First, data from the European Centre for Medium-Range Weather Forecasts (ECMWF) Interim Reanalysis^31^ (ERA-Interim) are mapped on the temperature–PWV surface. The domain is north of 70°N and 70°E to 220°E, corresponding roughly to the region where interannual variability in sea-ice conditions is largest (Supplementary Fig. 2). For each month, the 5-year running mean CRE anomalies are calculated using 1979–2000 as a baseline. Results are then scaled to account for the annual cycle in cloud fraction and optical depth. This scaling is fixed for all years such that cloud physical properties are held constant for reasonable modern values so that the resulting projected anomalies are attributed to changes in the temperature and opacity of water vapour. The anomaly time series for each month are plotted in Fig. 6c. This same calculation is then made using the Community Earth System Model Large Ensemble (CESM-LE)^32^ from 1979 through 2080, combining historical runs through 2005 and projections beginning in 2006 that use the Representative Concentration Pathway 8.5 (ref. 33; Fig. 6b,d).

Positive anomalies in CRE in both ERA-Interim and CESM-LE (Fig. 6c,d) emerge in autumn and early winter in the early 2000s. The mean anomaly for September through November from 2005 to 2012 is +2.5 W m^−2^ (ERA-Interim) and +2.7±1.6 W m^−2^ (CESM-LE; Supplementary Fig. 3). The largest anomalies are projected by CESM-LE to appear after 2040 in autumn between 5 and 15 W m^−2^. This result is associated with temperature increases in autumn outpacing the expected water vapour increases via the Clausius–Clapeyron relationship (red versus black lines in Fig. 6b). A similar signal is observed in spring, although it is smaller, in part because of less cloud cover and generally thinner clouds in that season. These model projections provide a conceptual understanding of how future changes in the Arctic system might have an impact on its sensitivity to CRE. In particular, the results for autumn likely represent a regime shift expressed as a change in the seasonal cycle of relative humidity that is driven by the increasing amount of open water in the Arctic. The consequence of these changes is that the feedback from clouds shown in Fig. 1 likely increases despite concomitant increases in absolute humidity.

Covariability between temperature and PWV in the Arctic does not closely fit the Clausius–Clapeyron relationship because of dominance by ice saturation at low temperatures and temperature buffering from latent energy exchange^21^ at 273 K. The latter results in relatively moist conditions for temperatures above 273 K, corresponding to lower CRE (that is, Figs 4 and 6b). For this reason, clouds in summer may contribute less flux in the future, by up to 3 W m^−2^, because of increasingly moist conditions for temperatures near 273 K in summer (Fig. 6b). Therefore, a relative dampening of the cloud feedback shown in Fig. 1 might be expected in summer.

## Discussion

This work presents the novel finding that, for a constant relative humidity profile, CRE_FIR_ and CRE_AW_ compensate each other as they change with temperature and PWV over ranges characteristic of the Arctic. The result has important implications in that the CRE remains fairly constant with warming as long as the relative humidity profile stays constant. This is in contrast to lower latitudes, where CRE is expected to decrease with increasing temperature at constant relative humidity, illustrating a unique sensitivity of the polar regions to climate change. In warmer, wetter conditions than the Arctic (for example, mid-latitudes and tropics, see Fig. 2), the FIR plays a negligible (rather than compensating) role, and CRE is thus dominated by CRE_AW_. Because CRE_AW_ decreases with increasing temperature and PWV, warmer/wetter regions are expected to become less sensitive to clouds with warming. In very cold, dry conditions (temperatures below ∼230 K), CRE_FIR_ is larger than CRE_AW_. CRE_AW_ increases with temperature and PWV, while CRE_FIR_ is transitioning similarly to the behaviour of CRE_AW_ near 280 K. When combined, the sensitivity to temperature is dominated by CRE_AW_, and thus CRE increases slightly with temperature and PWV. We speculate that below 230 K this may cause a positive feedback whereby increases in temperature increase CRE, which further increases temperature. Although the feedback is probably small, it is notable that it is driven by temperature alone.

In the Arctic, where changes in CRE_AW_ and CRE_FIR_ with temperature (at constant relative humidity) compensate, changes in covariability between temperature and humidity are necessary to modify CRE independently of variability in cloud properties. This work demonstrates this process both through radiative transfer calculations and through observations using spectrally resolved measurements; because similar instrumentation is available in multiple locations, we also observe how the process modulates the climate in different regions of the Arctic. Owing to the described mechanism, changes in the way temperature and moisture vary relative to each other cause shifts in the control of infrared irradiance between atmospheric gases and clouds, modifying the separation in downwelling infrared flux (W m^−2^) between the Arctic clear and cloudy states. The cloud feedback described in Fig. 1 is amplified or dampened accordingly, and, with it, the importance of variability in cloud microphysical and macrophysical properties. Indeed, using temperature and humidity from reanalysis data and climate model projections, we show trends of increasing CRE in the Arctic in all seasons except summer. Projected changes in Arctic cloud optical depth and cloud cover in autumn correspond to +3.8 and +14 W m^−2^ (2081–2100 minus 1961–1980)^5^ of infrared forcing, respectively, which is similar in magnitude to changes in CRE described here.

The variability in CRE described here is independent of shortwave CRE because the analysed perturbations are associated with atmospheric gases, which largely transmit solar radiation. This suggests a partial offset of the shortwave cooling supported by clouds and points to a reduction in the dampening of the ice-albedo feedback in the presence of increased cloud cover, a connection that is linked to interannual variability in sea ice^34^. Our findings also highlight the complexity of interpreting projections of cloud feedbacks. For example, decreases in cloud occurrence can accompany decreases in relative humidity, as was the case in summer 2007 (refs 23, 35), yet our results suggest that such changes could still result in net increases in CRE because the clouds that do form each contribute relatively more infrared flux to the surface energy budget.

ERA-Interim may be a more robust data set for climate monitoring than other reanalyses, including in the Arctic^19^,^36^, and the CESM-LE permits accounting for natural variability^32^. Nevertheless, caution must be taken when interpreting the time series shown in Fig. 6 because potential changes in the long-term temperature–humidity covariability are poorly understood. Regardless, the results presented here demonstrate conceptually the substantial sensitivity of CRE to modest, and realistic, shifts in the Arctic atmospheric state and provides further motivation for the need to reduce uncertainties in cloud properties in climate models. More research is also needed to interpret how temperature–humidity and temperature–humidity–CRE interactions occur over long timescales in association with external forcing and low-frequency natural variability. In addition, the associated enhancement (as is likely in autumn, winter and spring) or dampening (as is likely in summer) of cloud feedbacks should be investigated.

## Methods

### Data

Observations from Summit (July 2010 to August 2012, 72.58°N, 38.48°W, 3,210 m) are from the Integrated Characterization of Energy, Clouds, Atmospheric state and Precipitation at Summit (ICECAPS) project^20^. Barrow observations (January 2006 to December 2008, 71.325°N, 156.625°W, 8 m) are from the US Department of Energy Atmospheric Radiation Measurement (ARM) Program obtained at the North Slope of Alaska (NSA) site. ARM and ICECAPS data are available at http://www.arm.gov. The Eureka measurements (March 2006 to December 2008, 80.053°N, 86.417°W, 10 m) were supported by the NOAA Earth System Research Laboratory (ESRL) and the Canadian Network for the Detection of Arctic Change (CANDAC). Eureka data used in this study are archived by NOAA-ESRL, available at ftp://ftp1.esrl.noaa.gov/psd3/arctic/eureka/. Observational data may also be accessed through the International Arctic Systems for Observing the Atmosphere (IASOA) data portal (http://www.esrl.noaa.gov/psd/iasoa/). CESM-LE can be acquired from https://www.earthsystemgrid.org/home.htm. ERA-Interim data are archived by ECMWF and are available at http://apps.ecmwf.int/datasets/.

### Observed CRE

To determine the observed CRE, *F*↓_all-sky_ (equation (1)) is derived from radiances measured by Atmospheric Emitted Radiance Interferometers (AERI) at each station; because these measurements are at zenith and do not include the entire spectral range of interest, they are supplemented with radiative transfer calculations using collocated radiosoundings as input. *F*↓_clear-sky_ is estimated entirely from radiative transfer calculations (ref. 6 and references therein). To calculate the partial-band fluxes used in this study (CRE_FIR_ and CRE_AW_), integrations are performed over subranges for the FIR (10–630 cm^−1^) and AW (700–1,390 cm^−1^), similar to ref. 28. These ranges are chosen to correspond to the nearest band edges in the Rapid Radiative Transfer Model (RRTM)^37^. A small amount of CRE occurs at frequencies outside of the partial-band spectral regions used here; the residual is −0.6±1.2 W m^−2^. CRE is calculated using observations and radiative transfer modelling, which account for scattering, reflection, anisotropy and cloud thickness.

### Radiative transfer modelling

For deriving idealized CRE (Fig. 4a–c), radiosoundings from Barrow (2002–2012) and Summit (2010–2015) are supplemented with the sub-Arctic summer standard atmosphere^38^ between the radiosonde termination height and 60 km. Carbon dioxide (CO_2_) is set to 395 p.p.m. with a constant mixing ratio with height and mixing ratios of O_3_, N_2_O, CH_4_, CO and O_2_ are set to values from the standard atmosphere. Radiative transfer calculations for gaseous emission are performed using RRTM, and scattering and emission from the clouds are calculated using discrete-ordinate-method radiative transfer^39^. The surface and cloud are near thermodynamic equilibrium for most low-level overcast Arctic clouds, such that the cloud and surface temperatures are typically within ∼5 K of each other (and net surface infrared radiation is typically less than 20 W m^−2^ ref. 30). Thus, to reduce the influence of unrealistic combinations of clouds and atmospheric states, only samples when the surface temperature is within 5 K of the cloud temperature are retained (*n*=4,664).

For calculations of modelled CRE (Fig. 2, curves), the radiative transfer calculations are the same as described above but only account for absorption, while scattering is ignored. These calculations are performed using the sub-Arctic summer standard atmosphere^38^ with an optically thick cloud in the layer between 1 and 2 km; the temperature profile is shifted uniformly and PWV is calculated assuming constant relative humidity at all levels.

### Effective cloud emissivity

CRE, calculated for optically thick clouds, can be roughly converted to time-averaged CRE by multiplying by the cloud fraction and the mean cloud emissivity as *ɛ*_cloud,effective,*ν*_=(*R*↓_all-sky,*ν*_–*R*↓_clear-sky,*ν*_)/B_*ν*_(*T*_s_), where *R* is the radiance at zenith and *B*_*ν*_(*T*_s_) is the Planck function using the near-surface air temperature. The calculation is made using 900 cm^−1^ for wavenumber, *ν*. Atmospheric transmission between the surface and the cloud base and cloud anisotropy are neglected. These approximations result in a conservative estimate for the magnitude of the anomalies displayed in Fig. 6.

## Additional information

**How to cite this article:** Cox, C. J. *et al.* Humidity trends imply increased sensitivity to clouds in a warming Arctic. *Nat. Commun.* 6:10117 doi: 10.1038/ncomms10117 (2015).

## Supplementary Material

Supplementary Information Supplementary Figures 1-3, Supplementary References
