# Supplementary material for: Humidity trends imply increased sensitivity to clouds in a warming Arctic
Source: Nat Commun. 2015 Dec 10;6:10117. doi: 10.1038/ncomms10117 (PMC4682107; doi:10.1038/ncomms10117)
Supplement: Supplementary Information — Supplementary Figures 1-3, Supplementary References [file ncomms10117-s1.pdf]

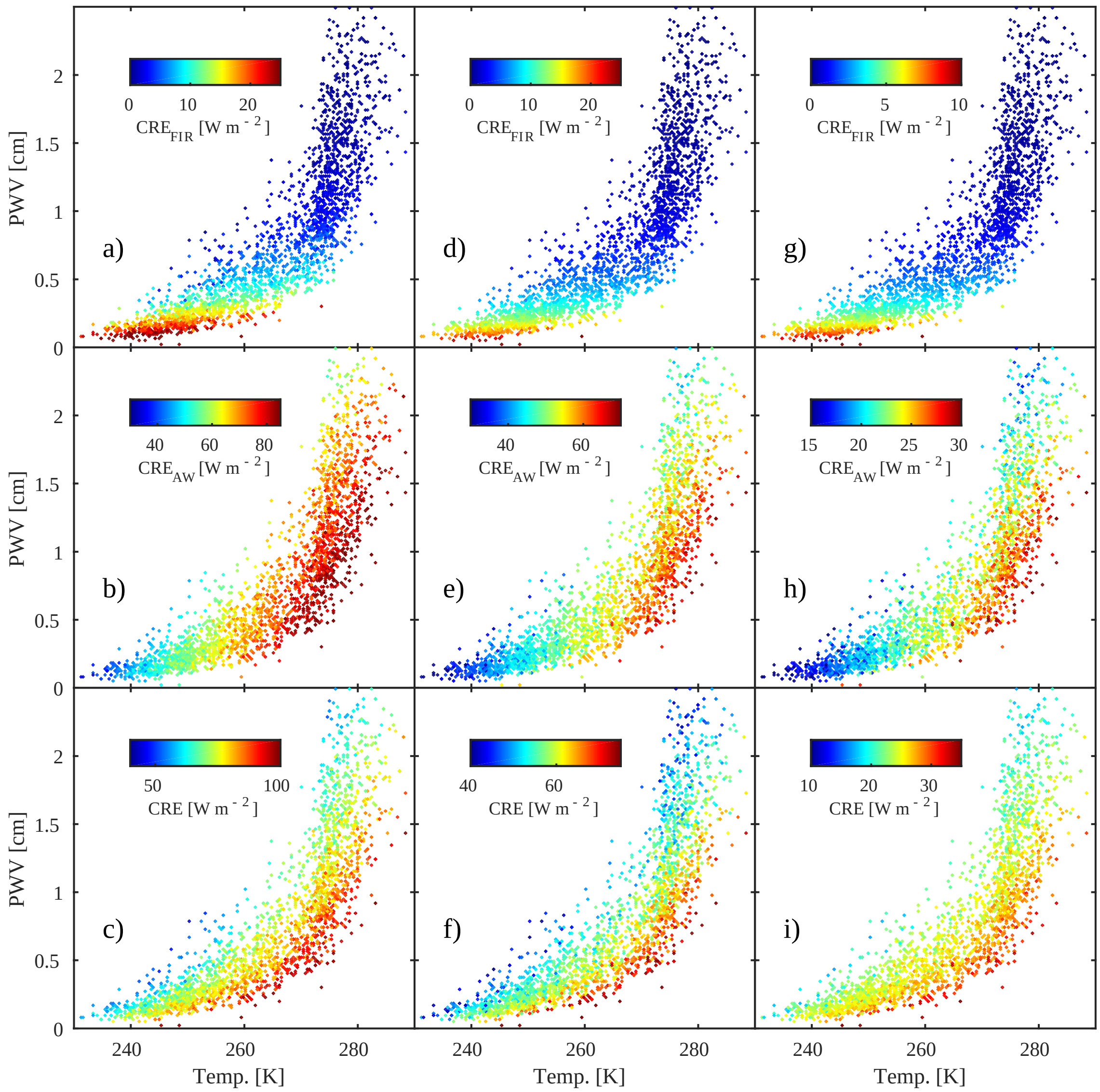

Supplementary Figure 1. Similar to Fig. 4 from the main text, but including clouds with smaller optical depths. Panels (a), (b) and (c) are for optical depths (τ) of 10; τ = 1 for panels (d), (e) and (f); and τ = 0.25 for panels (g), (h) and (i). Only calculations using radiosondes from Barrow, Alaska are shown.

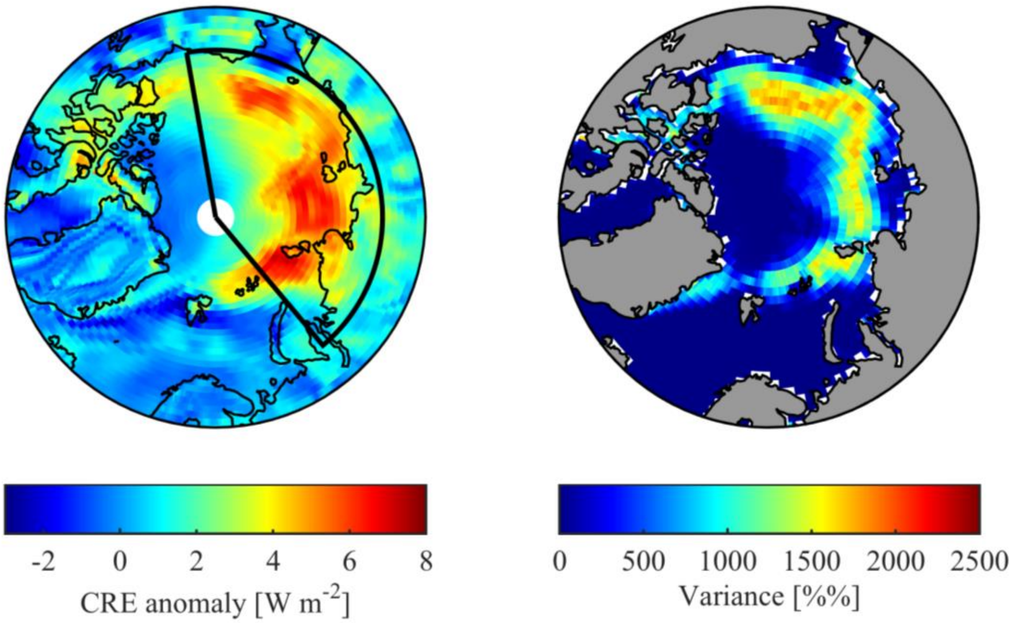

Supplementary Figure 2. (a) Spatial representation of an example year and month from Fig. 6c from the main text showing the estimated cloud radiative effect (CRE) anomaly from European Centre for Medium-Range Weather Forecasting Interim reanalysis (ERA-Interim) (ref. 1) for October 2012 based on 1979-2000 mean. CRE anomalies from ERA-Interim and the Community Earth System Model Large Ensemble (CESM-LE) (ref. 32) (Fig. 6d) are calculated north of 70ºN and from 70ºE to 220ºE, represented by the box in (a). (b) Variance in the mean September sea ice concentration from 1979 to 2014 from the Hadley Centre Sea Ice and Sea Surface Temperature data set (HadISST) (ref. 2).

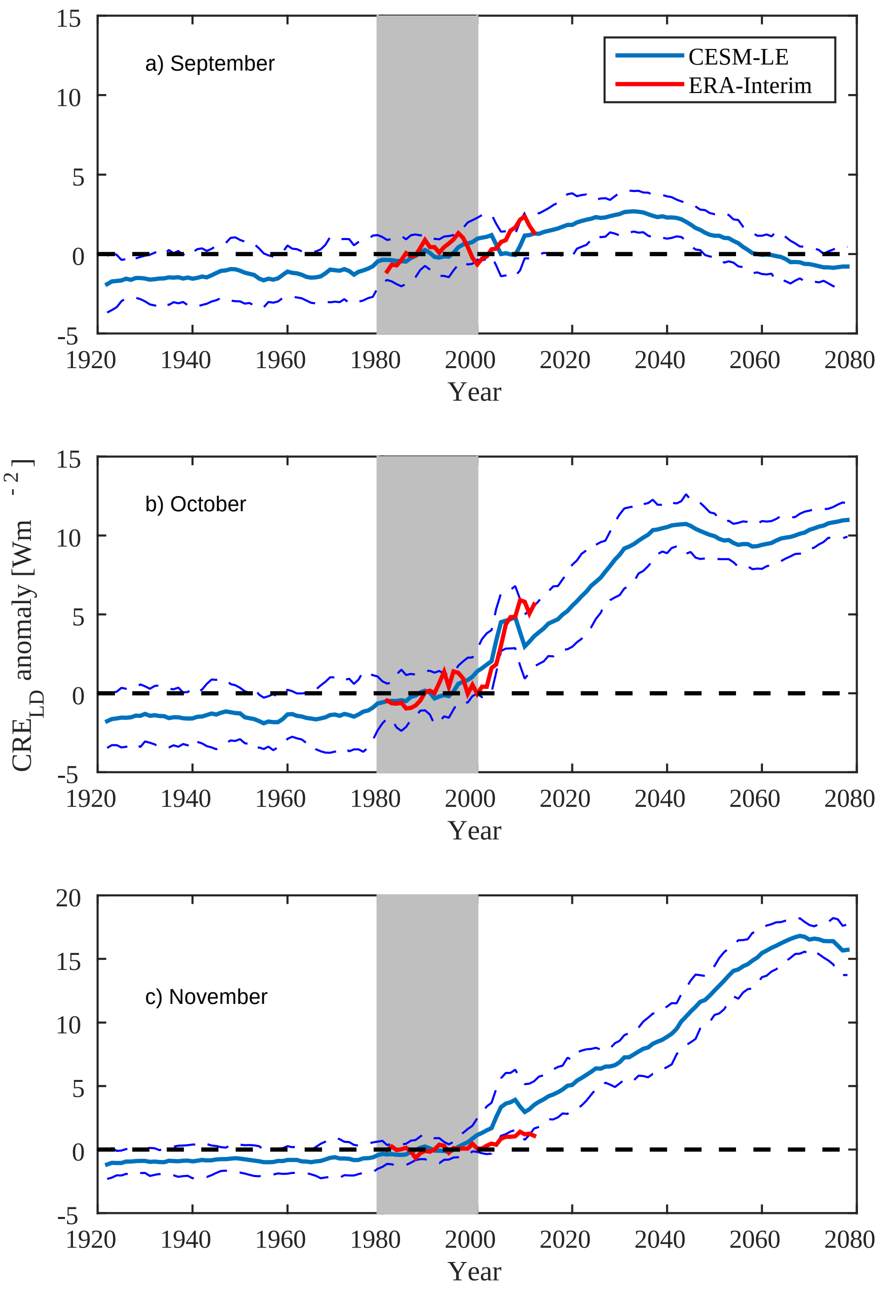

Supplementary Figure 3. European Centre for Medium-Range Weather Forecasting Interim reanalysis (ERA-Interim) (ref. 1) and Community Earth System Model Large Ensemble (CESM-LE) (ref. 3) estimated cloud radiative effect (CRE) anomalies from Figure 6c and 6d from the main text displayed as times series on the same axes for comparison: (a) September, (b) October, and (c) November. Dashed blue lines represent the ± 1σ variability amongst the thirty CESM-LE ensemble members. The grey box represents the 1979 to 2000 baseline from which the anomalies are calculated. Scaling for cloud fraction and optical depth is applied.

Supplementary References

1. Dee, D.P. et al. The ERA-Interim reanalysis: configuration and performance of the data assimilation system. *Q. J. Royal Meteorol. Soc.* **137**, 553-597 (2011).

2. Rayner, N.A. et al. Global analyses of sea surface temperature, sea ice, and night marine air temperature since the late nineteenth century. *J. Geophys. Res.* **108**, 4407 (2003).

3. Kay, J.E. et al. The Community Earth System Model (CESM) Large Ensemble Project: A community resource for studying climate change in the presence of internal climate variability. *Bull. Amer. Meteorol. Soc.* **96**, 1333-1349 (2015).
